# Impaired expression of CXCL5 and matrix metalloproteinases in the lungs of mice with high susceptibility to *Streptococcus pneumoniae* infection

**DOI:** 10.1002/iid3.205

**Published:** 2017-11-09

**Authors:** Rubia I. Mancuso, Eliane N. Miyaji, Cristiane C.F. Silva, Fernanda V. Portaro, Alessandra Soares‐Schanoski, Orlando G. Ribeiro, Maria Leonor S. Oliveira

**Affiliations:** ^1^ Laboratório de Bacteriologia Instituto Butantan São Paulo‐SP Brazil; ^2^ Laboratório de Imunoquímica Instituto Butantan São Paulo‐SP Brazil; ^3^ Laboratório de Imunogenética Instituto Butantan São Paulo‐SP Brazil

**Keywords:** inflammation, innate immunity, respiratory infections, *Streptococcus pneumoniae*

## Abstract

**Introduction:**

*Streptococcus pneumonia*e colonizes the nasopharynx of healthy individuals establishing a commensal relationship with the host. In some conditions, bacteria invade the lower respiratory tract and innate immune responses are crucial to avoid diseases such as pneumonia, sepsis, or meningitis.

**Methods:**

Here, we compared the susceptibility to pneumococcal respiratory infection of two outbred mouse lines, AIRmin and AIRmax, selected for low or high acute inflammatory responses, respectively.

**Results:**

AIRmin mice showed increased susceptibility to infection with different pneumococcal serotypes, when compared to AIRmax. Significant higher numbers of alveolar macrophages expressing the CD206 mannose receptor were observed in AIRmin mice when compared to AIRmax mice. Despite this difference, secretion of several cytokines and chemokines in the respiratory tract of AIRmin and AIRmax mice, after infection, was similar. The only exception was CXCL5, which was highly induced after pneumococcal infection in AIRmax mice but not in AIRmin mice. Reduced expression of the matrix metalloproteinases (MMP) 2, 3, 8, and 9, as well as reduced activities of MMPs were also observed in the lungs of AIRmin mice, after infection. Such impaired responses may have contributed to the low influx of neutrophils observed in the airways of these mice. Finally, high percentages of macrophages and neutrophils in apoptosis or necrosis, at the site of infection, were also observed in AIRmin mice, suggesting that leukocyte functionality is also compromised.

**Conclusions:**

Our results indicate that CXCL5 and MMPs contribute to the resistance to pneumococcal infection in mice.

## Introduction

*Streptococcus pneumoniae* (pneumococcus) is an important cause of diseases such as pneumonia, bacteremia and meningitis. It is estimated that around 400,000 children less than 5 years old die of pneumococcal diseases, per year, worldwide 1. Nevertheless, healthy individuals are commonly colonized by pneumococci and bacteria can persist in the nasopharynx for months without causing any harm. Inflammatory responses play an important role in the control of infection and drive the clearance of pneumococci from the nasal mucosa. Failure in this process can eventually lead to an increase in bacterial levels and promote invasion to the lungs and the bloodstream 2. Some conditions, such as ageing, polymorphisms in genes related to inflammation or even inflammatory burst caused by previous viral infections can predispose or compromise the recovery from disease 3, 4.

Some aspects of pneumococcal infection in humans can be reproduced in mice models. In mice lungs, alveolar macrophages (AM) represent the first line of defense against infection through the initial phagocytosis of bacteria and through the secretion of cytokines and chemokines that drive the recruitment of neutrophils 3. Recruited neutrophils will then be the major cells responsible for the clearance of bacteria through phagocytosis 2. Upon clearance, inflammatory signals decrease, reestablishing the basal levels of immune mediators and cells in the airways. Resolution of inflammation is also dependent on the apoptosis of AM and neutrophils and on the elimination of these cells by phagocytosis 5, 6, 7. BALB/c and CBA/c mice are commonly used in models of pneumococcal infection since BALB/c mice display a more resistant phenotype whereas CBA/c mice are more susceptible 8. Susceptibility of CBA/c mice was associated with a decreased capacity of TNF‐α secretion in the airways 9 and reduced recruitment of neutrophils to the site of infection 10. After intranasal infection, CBA/c mice display a continuous increase in bacterial loads in the lungs and invasion of bacteria to the bloodstream 9. In vitro, pneumococcal infection of bone marrow‐derived macrophages from CBA/Ca mice has also shown reduced secretion of TNF‐α and CCL5 and increased cell death when compared to BALB/c mice, suggesting that differences in macrophage responses may reflect in vivo susceptibility 11.

Here, we aimed to compare the susceptibility to pneumococcal infection of two outbred mouse lines, AIRmax and AIRmin, that were selected for high or low acute inflammatory responses (AIR), respectively. Animals were derived from a F0 founding population produced through the intercross of eight inbred strains. AIRmin and AIRmax mice were then established through bidirectional selective breeding on the basis of the AIR induced after subcutaneous injection of polyacrylamide beads, using cell counts, and protein concentration in inflammatory exudates as parameters 12. This model allowed the evaluation of pneumococcal susceptibility in animals with heterogeneous genetic backgrounds but homogeneous AIR, concerning the parameters mentioned above, due to combinations of gene alleles during selection. Our results support that differences in inflammatory responses can influence the outcome of pneumococcal infection and indicate that impaired expression of CXCL‐5 and matrix metalloproteinases is associated with susceptibility to invasive infection with a serotype 3 pneumococcal strain.

## Materials and Methods

### Mice

AIRmin and AIRmax lines (Ibut:AIRL and Ibut:AIRH formal stock designations at ILAR, Institute for Laboratory Animal Research, National Research Council, Washington DC), originate from the laboratory of Immunogenetics, Instituto Butantan (São Paulo, Brazil) 12. The experiments were carried out with 6‐ to 8‐weeks‐old male and female mice (4–6 mice per group) and groups were matched for gender. Female SPF BALB/c and C57BL/6 mice were produced by the animal facility from the Medical School of University of São Paulo, Brazil. Animals were supplied with food and water ad libitum. In experiments of lethal infections, humane endpoints were adopted and all animals displaying stages of disease that compromised activity or food acquisition were immediately euthanized. All procedures were performed in accordance to the guidelines of the Brazilian National Council for Control of Animal Experimentation (CONCEA) and were approved by the Ethic Committee on Animal Use from Instituto Butantan (license 1200/14).

### Bacterial strains and growth conditions

*S. pneumoniae* ATCC6303 (serotype 3), ATCC6301 (serotype 1), and M10 (serotype 11A) 13 strains were grown in Todd‐Hewitt broth (Difco Laboratories Inc., Detroit, MI, USA) supplemented with 0.5% yeast extract (THY) at 37°C without shaking. Bacteria were always plated on blood agar and grown at 37°C, before inoculation in THY. For challenge experiments, bacteria were grown until OD_600 nm_ = 0.4. Stocks were maintained at −80°C in THY containing 20% glycerol.

### Intranasal pneumococcal challenges and recovery of bacteria from lungs

Animals were anesthetized by intraperitoneal (i.p). route with 20 mg/Kg of xylazine and 50 mg/Kg of ketamine and received 3 × 10^4^ CFU of the ATCC6303 strain, 3 × 10^5^ CFU of the ATCC6301 strain, or 1 × 10^6^ CFU of the M10 strain in 50 μL of saline, inoculated into one nostril. In some experiments, mice were inoculated i.p. with the MMP inhibitors GM6001 or SB‐3CT (Calbiochem, San Diego, CA, USA), 2 h before the pneumococcal challenge and 24 h later. GM6001 and SB‐3CT doses of 50 and 25 mg/Kg, respectively, were chosen based on previous publications 14, 15. Both inhibitors were diluted in saline containing 10% DMSO and 10% Tween 20. Control groups received the vehicle alone. Animals were observed for 10 days for survival records or were euthanized at different time‐points with 60 mg/Kg of xylazine and 300 mg/Kg of ketamine for collection of Bronchoalveolar Lavage Fluids (BALF) or lungs as described before 16. Control samples were collected from mice that were not submitted to pneumococcal challenge. Serial dilutions of BALF and lung homogenates were plated on blood agar containing 4 μg/mL of gentamicin and CFU counting was performed after overnight incubation at 37°C. The minimal limit of detection was 120 CFU for BALF and 100 CFU for lungs.

### Characterization of cell populations and analysis of apoptosis

BALF were centrifuged at 100*g* for 10 min at 4°C and 1 × 10^5^ cells were stained with a mixture of anti‐F4/80 PE‐Cy7 (BM8, eBiosciences, San Diego, CA, USA), anti‐CD11b BB515 (M1/70), anti‐CD11c PE (HL3), anti‐Ly6G APC‐Cy7 (1A8), anti‐CD80 PerCP‐Cy5.5 (16‐10A1), and anti‐CD86‐APC (GL1) (Becton Dickinson, Franklin Lakes, NJ, USA) or a mixture of anti‐F4/80 PE‐Cy7, anti‐CD11b BB515, anti‐CD11c PE, anti‐Ly6G APC‐Cy7, and anti‐CD206 Alexa fluor 647 (MR5D3, AbD Serotec). Characterization of alveolar macrophages (AM), exudate macrophages (EM), and neutrophils were based on previous publications 17, 18. For the analysis of apoptosis 1 × 10^5^ cells were stained with a mixture of anti‐F4/80‐PE‐Cy7, anti‐Ly6G‐BV421 (1A8), anti CD11c‐BV510 (HL3), anti CD11b‐APC‐Cy7 (M1/70), Annexin V FITC, and 7‐AAD (Becton Dickinson). All antibodies were diluted in PBS containing 2% Fetal Calf Serum. Cells were evaluated in FACSCantoII (Becton Dinkinson) with 20,000 events recorded, with prior exclusion of low complexity populations through FSC and SSC parameters. Data were analyzed using the FlowJo V10 software.

### Detection of cytokines, chemokines, and MMPs

Cytokines, chemokines, and MMPs were detected in BALF using magnetic bead panel kits (Milliplex MAP, Merck Millipore, Burlington, MA, USA) for the presence of IFN‐γ, TNF‐α, IL‐1α, IL‐1β, IL‐2, IL‐4, IL‐5, IL‐6, IL‐7, IL‐9, IL‐10, IL‐12 (p40), IL‐12 (p70), IL‐13, IL‐15, IL‐17, G‐CSF, GM‐CSF, CXCL1, CXCL2, CXCL10, CCL2, CCL3, CCL4, CCL5 (MCYTOMAG‐70K‐PMX) or MMP‐2, MMP‐3, MMP‐8, proMMP‐9, and MMP‐12 (MMP3MAG‐79K). Proteins were detected in the Luminex MAGPIX system equipment (Merck Millipore) using the Xponent software (Luminex, Austin, TX, USA). Data were analyzed with the Milliplex Analyst software (Merck Millipore), based on standard curves plotted through a 5‐parameter logistic curve setting. Detection of CXCL5 was performed by ELISA of BALF samples, using the mouse LIX DuoSet ELISA kit (R&D Systems Minneapolis, MN, USA) according to the instructions of the manufacturer.

### Analyses of MMPs activity

Enzymatic activity was measured in BALF using a fluorescent resonance energy transfer (FRET) substrate, Abz‐AGLA‐EDDnp 19, 20. Abz‐AGLA‐EDDnp was synthesized using automated solid‐phase synthesis (GenOne Biotechnologies, RJ, Brazil) and was kindly provided by Dr. Ana Maria Moura da Silva (Immunopathology Laboratory, Butantan Institute). The substrate was chosen due to MMPs primary specificity GP(A/G)G↓L(A/R)G 21. BALFs (20 μL) from AIRmin and AIRmax mice, collected at 6 and 12 h post‐challenge, were incubated with 5 µM of Abz‐AGLA‐EDDnp at 37°C in PBS, pH 7.4. For MMPs inhibition, 20 μL of BALFs were incubated with 10 µM of GM6001 or SB‐3CT at room temperature for 30 min before reaction. Control reactions were performed in the presence of an equal volume of DMSO (used to solubilize the inhibitors). Assays were performed in triplicate and the specific peptidase activities were expressed as unit of free fluorescent of the cleaved substrate per minute per µL of BALF (UF/min**/**µL). Reactions were monitored in a fluorimeter (Victor 3™, Perkin‐Elmer, Whaltam, MA, USA) at λ _EM_ 420 nm and λ _EX_ 320 nm.

### Statistical analysis

Differences in survival were analyzed by Log‐Rank test. Differences in CFU were analyzed with the Unpaired *T* test or the two‐way analysis of variance (ANOVA), according to the experiment. Differences in the numbers of cells, cytokines, chemokines, and MMPs expression and MMPs activities were also analyzed by two‐way ANOVA. In all cases Tukey's post‐test was used for comparisons between groups. Analyses were performed using Prism 6 GraphPad, and *P* values ≤0.05 were considered significantly different.

## Results

### AIRmin mice are more susceptible to pneumococcal infection than AIRmax mice

**Figure 1:**
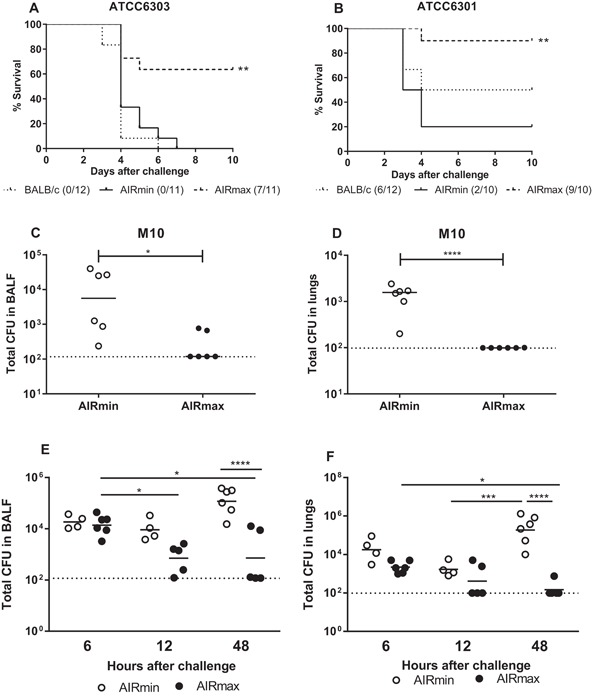
AIRmin mice are more susceptible to pneumococcal infection than AIRmax mice. Animals were submitted to intranasal pneumococcal challenge with the serotype 3 ATCC6303 (A, E, and F), the serotype 1 ATCC6301 (B) or the serotype 11A M10 strains (C and D). In A and B, survival of mice was monitored for 10 days. **p < 0.01, Log‐Rank survival curve (Comparison between AIRmin and AIRmax). Curves were composed with the results of two independent experiments. Numbers indicate alive/total animals in each group. In C and D mice (6 per group) were euthanized 24 h post‐infection; in E and F mice (4 to 6 per group) were euthanized at different time‐points post‐infection and bacteria were recovered from BALF or lungs. Circles represent each individual and lines represent the medians of the groups. *p < 0.05, **p < 0.01, ***p < 0.001, Unpaired *T* test (C and D) and Two‐way ANOVA, with Tukey's post‐test (E and F). Results from C, D, E and F represent at least two independent experiments.

AIRmin and AIRmax mice were submitted to respiratory challenges with different pneumococcal serotypes. Infection of mice with the ATCC6303 strain (serotype 3), induced the death of 100% of AIRmin mice, a result similar to the observed for BALB/c mice. The same dose of bacteria induced the death of only 36.4% of AIRmax mice (Fig. 1A). Challenge of mice with the ATCC6301 strain (serotype 1), which has shown a less virulent phenotype in BALB/c mice, killing 50% of the animals, resulted in the death of 80% of AIRmin mice, but only 10% of AIRmax mice (Fig. 1B). Furthermore, using a model of non‐lethal infection with a serotype 11A strain, it was possible to recover higher numbers of pneumococci, at 24 h post‐challenge, from BALF and lungs of AIRmin mice when compared to AIRmax mice (Fig. 1C,D). The susceptibility of AIRmin and AIRmax mice was also compared using a nasal colonization model with a serotype 6B strain. However, no significant differences in nasal colonization were observed at day 5 or 7 post‐infection (Fig. S1).

Since differences in susceptibility were observed for the lung infection challenges, the ATCC6303 challenge model was chosen for the next experiments. Evaluation at different time points post‐challenge with the ATCC6303 pneumococcal strain, showed an increase in total numbers of bacteria at 48 h in AIRmin mice, both in BALF and lungs. On the other hand, bacterial numbers progressively decreased in the respiratory tract of AIRmax mice. At 48 h post‐infection, the levels of bacteria were significant higher in AIRmin mice when compared to the observed in AIRmax mice (Fig. 1E,F). Since few AIRmax mice could not control infection, only mice that showed undetectable levels of bacteria in the lungs at 48 h were considered for comparative analyses of immune responses at this time point. For the rest of the time‐points, all animals were included in the analyses.

### Pneumococcal infection leads to a strong influx of neutrophils in the respiratory tract of AIRmax mice

**Figure 2:**
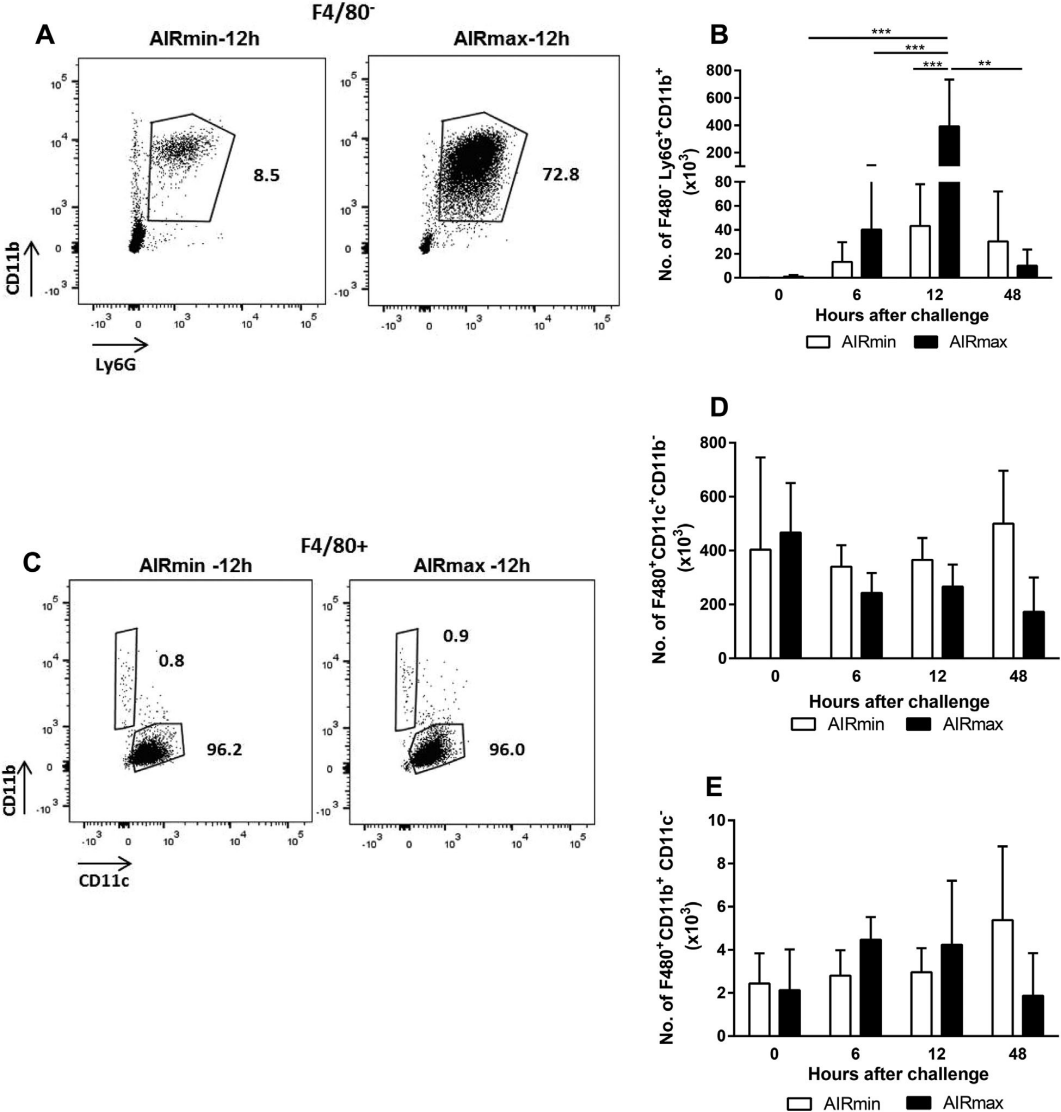
Pneumococcal challenge induces the influx of neutrophils to the respiratory tract of mice with higher intensity in AIRmax mice. Animals (4 to 6 per group) were submitted to intranasal pneumococcal challenge with the ATCC6303 strain and were euthanized at different time‐points post‐challenge. Total cells from BALF were stained for the evaluation of neutrophils (A and B), AM (C and D), and EM (C and E) by flow cytometry. A and C are representative flow plots of the neutrophil (F4/80^−^ CD11b^+^ Ly6G^+^) AM (F4/80^+^ CD11c^+^ CD11b^−^) and EM (F4/80^+^ CD11b^+^ CD11c^−^) populations, respectively, at 12 h post‐challenge. Numbers indicate the percentages inside the F4/80^−^ (A) or the F4/80^+^ (C) populations. Graphs show total number of neutrophils (B), AM (D), and EM (E) in BALF at each time point post‐challenge. Results were expressed by means for each group with SD and are representative of two independent experiments. ***p < 0.001, Two‐way ANOVA, with Tukey's post‐test.

BALF were collected before (0 h) and at different time‐points post‐challenge and the populations of cells (selected according to the strategy shown in Fig. S2) were compared by flow cytometry. The percentage of neutrophils (F4/80^−^ Ly6G^+^ CD11b^+^) was higher in AIRmax mice at 12 h post‐challenge, when compared to AIRmin mice (Fig. 2A, representative flow plot). Analysis of total numbers showed a strong increase in neutrophils at 12 h post‐challenge in AIRmax mice (reaching around 4 × 10^5^ cells per BALF), with significant differences when compared with the same time‐point in AIRmin mice (4 × 10^4^ cells/BALF). The numbers of neutrophils were reduced to almost the basal levels in AIRmax mice that controlled infection at 48 h (Fig. 2B).

Alveolar macrophages (AM), immunophenotyped as F4/80^+^ CD11c^+^ CD11b^−^, were the majority of the F4/80^+^ cells in the respiratory tract of mice before (0 h) and after the challenge (Fig. 2C, representative flow plot). No differences were observed in total numbers of AM (around 2 × 10^5^ to 4 × 10^5^ cells per BALF) in the airways of AIRmin and AIRmax mice after the pneumococcal challenge (Fig. 2D). Additionally, pneumococcal challenge did not induce the influx of exudate macrophages (EM), F4/80^+^ CD11b^+^ CD11c^−^, to the airways of mice (Fig. 2E). This population represented the minority of the F4/80^+^ cells in all time‐points (around 2 × 10^3^ to 4 × 10^3^ per BALF) (Fig. 2C, representative flow plot).

### Increased numbers of AM expressing the CD206 mannose receptor were observed in AIRmin mice

**Figure 3:**
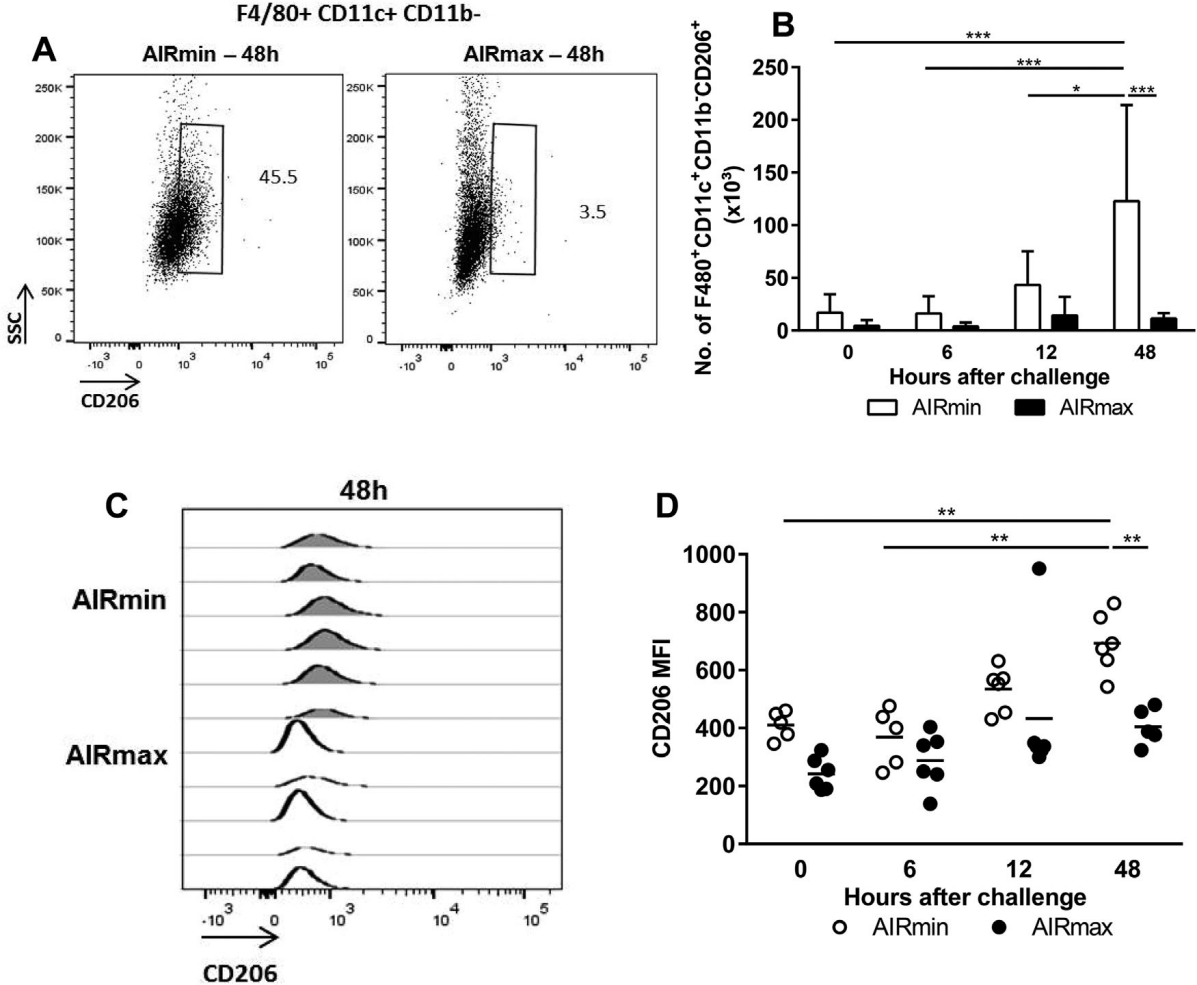
AIRmin mice showed an increase in AM expressing the CD206 receptor. Animals (5 to 6 per group) were submitted to intranasal pneumococcal challenge with the ATCC6303 strain and were euthanized at different time points post‐challenge. Total cells from BALF were stained for the evaluation of AM expressing CD206 (F4/80^+^ CD11b^+^ CD11c^−^ CD206^+^) by flow cytometry. (A) Representative flow plots of the CD206^+^ alveolar macrophages populations at 48 h post‐challenge. Numbers indicate the percentages inside the F4/80^+^ CD11b^+^ CD11c^−^ population. (B) Total number of AM expressing CD206. Results were expressed by means for each group with the standard deviations. (C and D) Expression of CD206 analyzed by the MIF displayed by the alveolar macrophage population. *p < 0.05; **p < 0.01; ***p < 0.001, and ****p < 0.0001, Two‐way ANOVA with Tukey's post‐test.

The AM population in response to pneumococcal challenge was characterized for the expression of the surface markers CD206, CD80, and CD86. AIRmin mice showed a significant increase in total numbers of CD206^+^ AM, especially at 48 h post‐challenge (Fig. 3B), when these animals displayed high bacterial burden. Total numbers of CD206^+^ AM were significantly higher than the observed in AIRmax mice at this time‐point (1 × 10^5^ cels/BALF were observed in AIRmin mice whereas AIRmax mice displayed 1 × 10^4^ cells/BALF). Evaluation of the mean fluorescence intensity (MFI) for CD206 staining showed that AM from AIRmin mice expressed more CD206 molecules on the surface, when compared to AM from AIRmax mice, particularly at 48 h post‐challenge (Fig. 3C,D). Total numbers of CD80^+^ or CD86^+^ AM remained unaltered in response to pneumococcal challenge both in AIRmin and AIRmax mice, but a trend to increase in the CD86^+^ population could be observed at late time‐points in both mice lines (not shown).

### Induction of cytokines and chemokines in response to pneumococcal challenge

**Figure 4:**
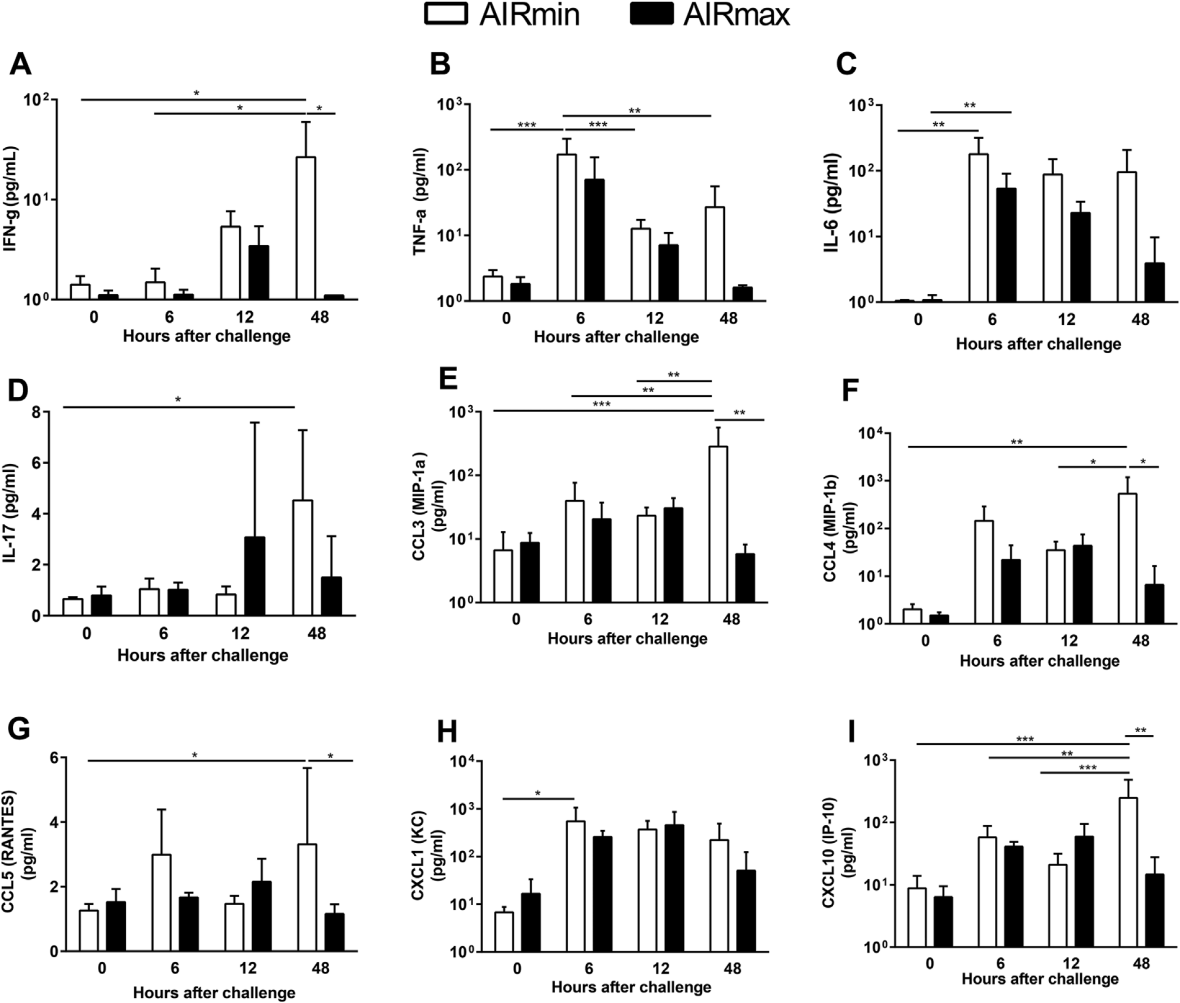
Cytokines and chemokines were induced in response to pneumococcal challenge in both AIRmin and AIRmax mice. Animals (4 to 6 per group) were submitted to intranasal pneumococcal challenge with ATCC6303 strain. Mice were euthanized at different time‐points post‐challenge and BALF were collected for the analysis of cytokines and chemokines by Luminex. Results were expressed by means for each group with SD and are representative of two independent experiments. *p < 0.05; **p < 0.01; ***p < 0.001 Two‐way ANOVA with Tukey's post‐test.

The differences in the population of AM led us to analyze the cytokine and chemokine responses. An increase in the secretion of the pro‐inflammatory cytokines IFN‐γ, TNF‐α, and IL‐6 was observed after pneumococcal challenge in both mice lines. As expected, the levels of these cytokines decreased at 48 h post‐challenge, in AIRmax mice that controlled infection (Fig. 4A–C). Timing and the magnitude of the cytokine responses in AIRmin mice were similar to the observed for AIRmax mice. However, such responses were not sufficient to control pneumococcal loads in these animals (Fig. 1) and the levels of IFN‐γ, TNF‐α, and IL‐6 remained high at late time‐points (Fig. 4A–C). Secretion of IL‐17 was highly variable among the animals and a significant increase was only observed for AIRmin mice at 48 h post‐challenge (Fig. 4D). Again, this response parallels with high pneumococcal loads in the lungs. Similar results were observed for the chemokines CCL3 (MIP‐1α), CCL4 (MIP‐1β) CCL5 (RANTES), and CXCL10 (IP‐10) (Fig. 4E–G,I), which were induced by the pneumococcal challenge in both mice lines and, at 48 h post‐challenge, were significantly higher in AIRmin mice than in AIRmax mice. Interestingly, although the magnitude of neutrophil influx in response to pneumococcal challenge was different between AIRmin and AIRmax mice, with high numbers observed in the latter (Fig. 2B), no differences were observed for the secretion of CXCL1 (KC), a known chemoattractant of these cells (Fig. 4H).

For the Th2 cytokines, IL‐4 and IL‐5, and the anti‐inflammatory cytokine IL‐10, the response to pneumococcal challenge peaked at 12 h, but returned to basal levels in both mice lines (Fig. S3A, B, and C). It is interesting to notice that a high variability in the levels of these cytokines at 12 h post‐challenge was observed for AIRmax mice, which is in accordance with the fact that not all animals respond equally to pneumococcal infection. Unfortunately, at this time point, it was impossible to determine the outcome to pneumococcal infection for each AIRmax mouse. The cytokines G‐CSF and GM‐CSF and the chemokines CCL2 (MCP‐1) and CXCL2 (MIP‐2) were also induced after the challenge with no differences between the two stocks of mice (Fig. S3D–G). Finally, no significant levels of IL‐13, a cytokine that is related to the induction of alternative activated macrophages, which usually express the CD206 receptor, was observed for both AIRmin and AIRmax mice (not shown). All other cytokines tested by Luminex showed levels below the limit of detection or the results could not be reproduced in independent experiments.

**Figure 5:**
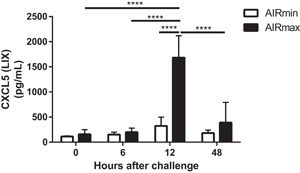
Pneumococcal infection induced the secretion of CXCL5 in the respiratory tract of AIRmax mice but not in AIR min mice. Animals (4 to 6 per group) were submitted to intranasal pneumococcal challenge with ATCC6303 strain. Mice were euthanized at different time‐points post‐challenge and BALF were collected for the analysis of CXCL5 by ELISA. Results were expressed by means for each group with SD and are representative of two independent experiments. *p < 0.05; **p < 0.01; ***p < 0.001 Two‐way ANOVA with Tukey's post‐test.

Since no significant differences were observed in the responses of AIRmin and AIRmax mice for all these immune mediators, we decided to analyze the secretion of CXCL5, a chemokine that was previously implicated in neutrophil influx in pneumococcal respiratory infection models in mice. The results obtained by ELISA showed a remarkable difference in the response to pneumococcal infection between the two mice lines (Fig. 5). Induction of high levels of CXCL5 was observed in AIRmax mice at 12 h after pneumococcal infection and the levels of this chemokine reduced at 48 h post‐infection, in association with the reduction in bacterial burden. On the other hand, AIRmin mice showed only basal levels of this chemokine in all time points evaluated (Fig. 5).

### Impaired expression of matrix metalloproteinases (MMPs), in response to pneumococcal infection, in the susceptible AIRmin mice

**Figure 6:**
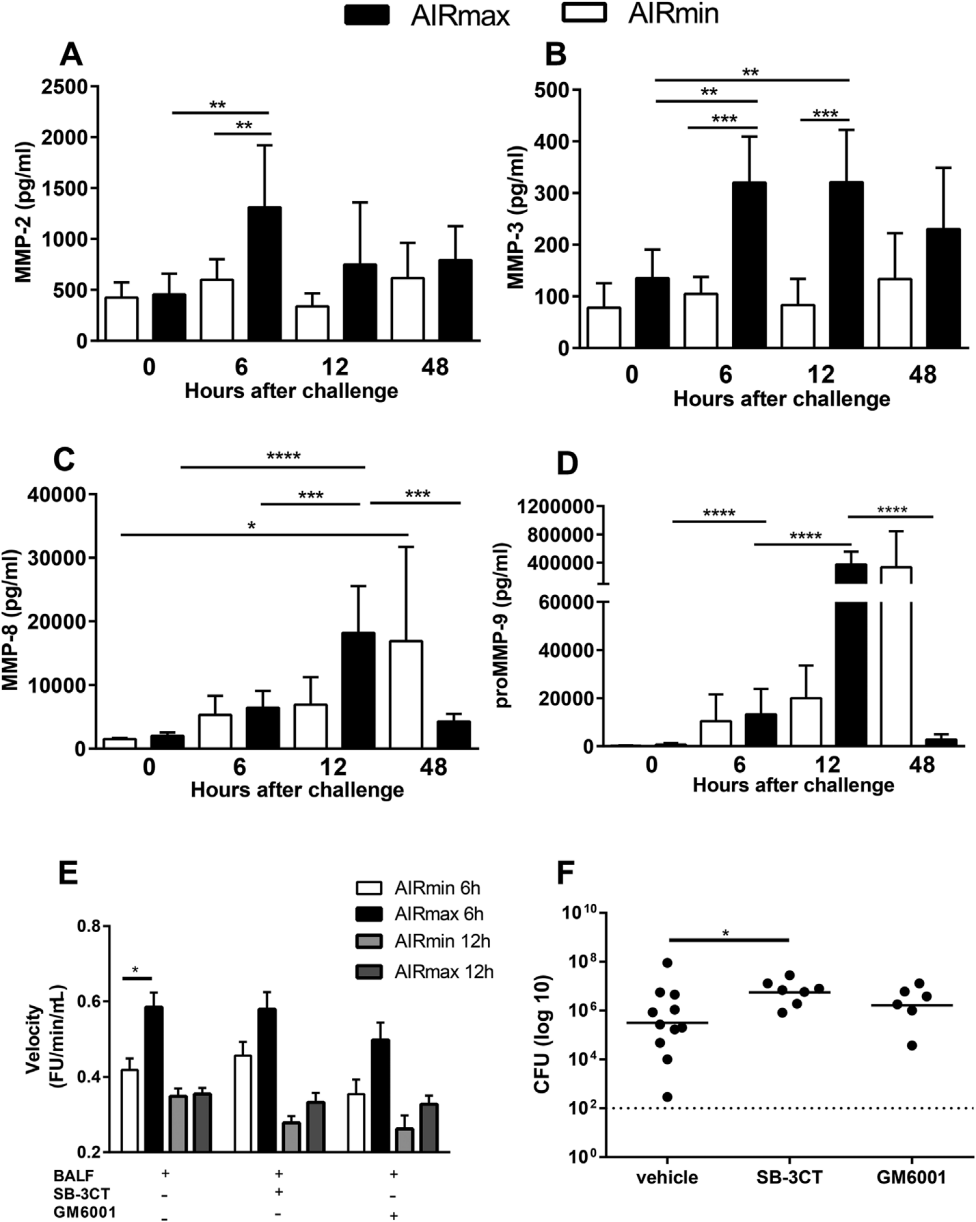
Expression and activity of MMPs, in response to pneumococcal challenge, are impaired in AIRmin mice. Animals (4 to 6 per group) were submitted to intranasal pneumococcal challenge with the ATCC6303 strain. (A–D) Mice were euthanized at different time‐points post‐challenge and BALF were collected for the analysis of MMPs by luminex. Results were expressed by means for each group with SD and are representative of two independent experiments. (E) Enzymatic activity was measured in BALF (from 8 mice per group) collected at 6 and 12 h post‐challenge, using a FRET substrate, Abz‐AGLA‐EDDnp. MMP inhibition was tested by previous incubation of BALF with GM6001 or SB‐3CT. BALF from two independent experiments were tested and reactions were performed in triplicates. Results were expressed by means of each group with SEM. (F) Mice were inoculated with the MMP inhibitors GM6001 or SB‐3CT 2 h before the pneumococcal challenge and 24 h later. Mice were euthanized at 48 h post‐challenge and CFU was evaluated by plating lung macerates on blood‐agar. Graphs were composed with results of two independent experiments. Circles represent each individual and lines represent the medians of the groups. *p < 0.05; **p < 0.01; ***p < 0.001, and ****p< 0.0001 (A–D) Two‐way ANOVA with Tukey's post‐test, (E) Two‐way ANOVA with Tukey's post‐test, (F) Unpaired *T* test.

MMPs expression in BALF was also analyzed by Luminex. A strong induction of MMP‐2 and MMP‐3 MMP‐8 and proMMP‐9, at 6 and 12 h post‐challenge, was observed in the airways of AIRmax mice. The levels decreased at 48 h post‐challenge, in parallel with the reduction in bacterial burden and with the control of inflammation. Conversely, pneumococcal infection did not induce the expression of MMP‐2 and MMP‐3 in AIRmin mice, not even at 48 h post‐challenge, when high numbers of bacteria were observed. MMP‐8 and proMMP‐9 showed a delayed expression, with high levels of observed only at 48 h post‐challenge (Fig. 6A–D). The activity of MMPs in BALF was tested using Abz‐AGLA‐EDDnp as substrate. Significant higher activities were observed at 6h post‐challenge in BALFs from AIRmax mice when compared to BALFs from AIRmin mice. However, no differences in activity were observed at 12 h post‐challenge. Thus, the differences in the expression of some MMPs observed at 12 h post‐challenge, did not reflect in differences in the hydrolysis of Abz‐AGLA‐EDDnp. Partial inhibition of the enzymatic activity was observed after previous incubation of BALFs with GM6001, a broad‐spectrum inhibitor (able to affect the activity of MMP‐1, MMP2, MMP3, MMP‐8, and MMP‐9) or SB‐3CT, a more specific inhibitor of MMP‐2 and MMP‐9. Inhibition of substrate hydrolysis was around 15% by GM6001 at 6h post‐challenge for both mice lines and 24% and 7% at 12 h post‐challenge for AIRmin and AIRmax mice, respectively. Reduction of substrate hydrolysis by the SB‐3CT inhibitor was only observed in samples collected 12 h post‐challenge, with decreases of 20% and 6% in AIRmin and AIRmax mice, respectively (Fig. 6E).

### Treatment with MMPs inhibitors affects bacterial clearance in AIRmax mice

The influence of MMPs activities on the resistance of AIRmax mice to pneumococcal infection, was evaluated by the treatment with GM6001 or SB‐3CT, 2 h before pneumococcal infection and 24 h after the first inoculation. Since no significant differences were observed in the health status of mice treated with the inhibitors at 48 h post‐challenge, animals were euthanized and the levels of bacteria in the lungs were compared. The groups treated with the MMP inhibitors showed more homogeneous levels of pneumococci in the lungs with all animals displaying high numbers of bacteria. Significant differences were observed for the group inoculated with the SB‐3CT when compared to the vehicle alone, indicating that the treatment produced a negative effect in the clearance of bacteria (Fig. 6F).

### High percentages of macrophages and neutrophils in apoptosis were observed in the respiratory tract of AIRmin mice

**Figure 7:**
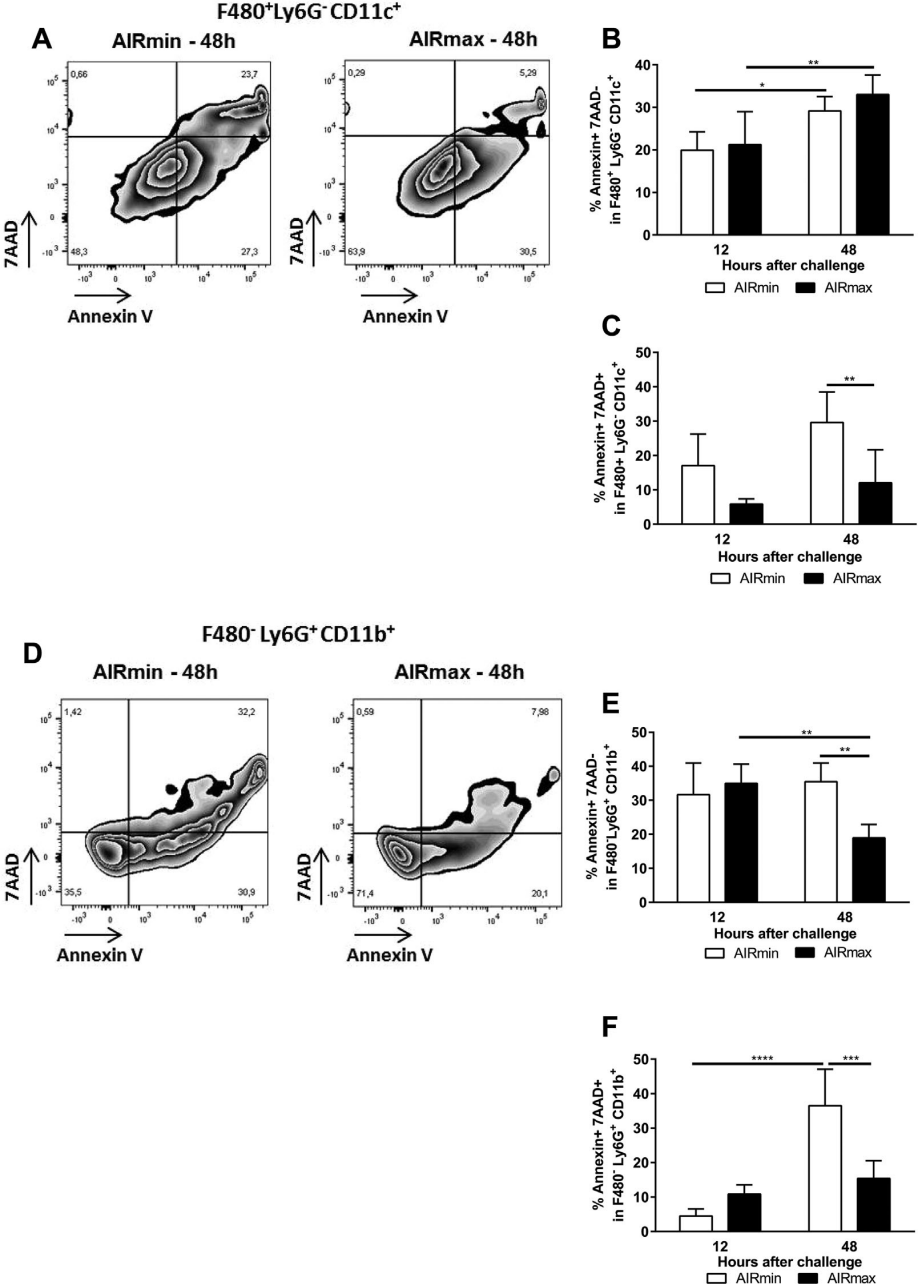
AIRmin mice showed higher percentages of apoptotic or necrotic macrophages and neutrophils in response to pneumococcal challenge. Animals (5 to 6 per group) were challenged with the ATCC6303 strain and were euthanized at different time‐points. Total BALF cells were stained for the evaluation of apoptosis in macrophages or neutrophils by flow cytometry. (A and D) Representative flow plots of macrophages (F4/80^+^Ly6G^−^ CD11c^+^) and neutrophils (F4/80^−^Ly6G^+^ CD11b^+^) at 48 h post‐challenge. Numbers indicate the percentages inside the populations. (B, C and E, and F) Percentages of macrophages or neutrophils in apoptosis (Annexin V^+^ 7AAD^−^) or necrosis (Annexin V^+^ 7AAD^+^). Results were expressed by means for each group with SD and are representative of two independent experiments. *p < 0.05; **p < 0.01; ***p < 0.001, Two‐way ANOVA with Tukey's post‐test.

Apoptosis of phagocytes is an important step for the clearance of pneumococci from lungs. Viability of macrophages from both mice stocks was similar before the challenge (data not shown). Percentages of macrophages (F4/80^+^ Ly6G^−^ CD11c^+^) in apoptosis (annexin V^+^/7AAD^−^) were also similar in AIRmin and AIRmax mice after the challenge (Fig. 7A, representative flow plot and Fig. 7B). However, AIRmin mice showed higher percentages of macrophages in necrosis (annexin V^+^/7AAD^+^) at 48 h post‐challenge, when compared to AIRmax mice (Fig. 7A,C). In addition, percentages of neutrophils (Ly6G^+^ F4/80^−^ CD11b^+^) in apoptosis or necrosis were higher AIRmin mice, when compared to AIRmax mice, at 48 h post‐challenge (Fig. 7D, representative flow plot and Fig. 7E,F).

## Discussion

The influence of AIR on pneumococcal infection was analyzed in this work using the two outbred AIRmin and AIRmax mouse lines. Despite the heterogeneous genetic background, AIRmin responses to pneumococcal infections were highly homogeneous, with almost all animals showing susceptibility to three different pneumococcal serotypes. Furthermore, susceptibility was observed for invasive respiratory infection and a restricted lung infection model. Our results thus show that susceptibility to pneumococcal infection was enhanced during AIRmin selection for low acute inflammatory responses. In contrast, AIRmax mice were significantly more resistant to pneumococcal infection when compared to both AIRmin and BALB/c mice. Most of these animals cleared bacteria from the lungs in around 48 h post‐challenge. In accordance to previous works using inbred mice strains 8, 10 resistance to pneumococcal infection was associated with a strong infiltration of neutrophils, observed at 12 h post‐challenge in AIRmax mice. Infiltration of neutrophils in response to pneumococcal infection was also observed in AIRmin mice, but the levels were significantly lower when compared to AIRmax mice. In fact, increased production of neutrophils by bone marrow, high numbers of circulating neutrophils and accelerated neutrophil maturation were shown to be convergent characteristics selected in AIRmax mice that may confer resistance to infections 22.

In models of resolving pulmonary infection, with low doses of pneumococci, AM play a central role on bacterial clearance through phagocytosis 7. In contrast, in high‐doses inoculums or in models of invasive pneumococcal infection, depletion of AM resulted in the increase of inflammatory cytokines and prolonged accumulation of polymorphonuclear cells in the lungs. In these cases, prolonged inflammation was associated with mortality 5, 23. In our model, we did not observe significant variations in total numbers of AM or exudate macrophages during pneumococcal infection in both AIRmin and AIRmax mice. Still, AIRmin mice displayed increased levels of CD206^+^ AM. CD206 is normally expressed by mice AM^24^. Some inflammatory conditions, can favor the polarization of macrophages to an alternative activation program (AAM or M2 macrophages) which is characterized by high expression of CD206 and secretion of Th‐2 cytokines such as IL‐4 and IL‐13 25, 26. AAM and the secretion of IL‐4 and IL‐13 have also been implicated in the increased susceptibility of mice to a secondary pneumococcal infection during recovery of influenza infection 27. However, despite the differences in CD206 expression, increased susceptibility of AIRmin mice to the pneumococcal challenge could not be associated with significant differences in the secretion of 25 cytokines or chemokines evaluated, including IL‐4 (Fig. S3) and IL‐13, which was below the limit of detection (data not shown). Secretion of IL‐10, an anti‐inflammatory cytokine associated with another type of M2 macrophages (Regulatory Macrophages) was also similar in both mice lines. In fact, pneumococcal challenge in AIRmin and AIRmax mice induced the secretion of pro‐inflammatory cytokines, such as TNF‐α, IFN‐γ, and IL‐6, which are characteristic of M1 macrophages. Therefore, no clear pattern of macrophage responses could be defined in our model. Increasing data on the complexity and the plasticity of macrophages responses come from in vivo experiments with different stimuli, showing that simultaneous expression of genes related to M1 and M2 macrophages can occur 28, 29.

Secretion of several cytokines and chemokines was comparable between AIRmin and AIRmax mice, both in intensity and in timing, with peaks around 6 and 12 h. Significant differences were only observed at 48 h post‐challenge, when these cytokines were reduced in AIRmax, correlating with the decrease in bacterial loads. Conversely, these mediators remained high in AIRmin mice in association with increasing numbers of bacteria, suggesting an uncontrolled stage of infection and inflammation in which tissue damage may occur and contribute to bacterial spread. This was observed for the secretion of IFN‐γ, IL‐6, and IL‐17, CCL3 (MIP‐1α), CCL4 (MIP‐1β) CCL5 (RANTES), and CXCL10 (IP‐10). Several of these mediators have already been implicated in the immune responses against *S. pneumoniae* in in vitro systems as well as in mice and humans. Th1 and Th17 responses are known to be effective against pneumococcal infections 30, 31. MIP‐1α and RANTES were shown to participate in macrophage infiltration to the lungs in a pneumonia model in CD1 mice 32. IP‐10 was recently associated with the pathogenesis of pneumonia, since elevated levels of this cytokine were observed in the sera of children with severe pneumonia due to mixed viral/ bacterial infections, including pneumococcal infections 33. Finally, secretion of CXCL1 (KC) and CXCL2 (MIP‐2) precedes neutrophil recruitment in a model of pneumococcal pneumonia in mice 32. This was also observed for AIRmin and AIRmax mice, although the influx of neutrophils to the airways was significant higher in the latter. Altogether, these observations implicate that the secretion of several inflammatory mediators in the respiratory tract of AIRmin mice was not sufficient to confer protection to against pneumococcal infection.

CXCL5 was the only chemokine that showed a diverse pattern of expression in AIRmin and AIRmax responses to pneumococcal infection. Whereas pneumococcal infection induced the secretion of high levels of CXCL5 in the airways of AIRmax mice, no response was observed in AIRmin mice. Yamamoto and collaborators have previously shown that CXCL5 is expressed by lung epithelial cells (EC) in response to pneumococcal infection. CXCL5 expression was dependent on Rel A activity in EC and the peak of mRNA was observed after 9h post‐infection in their model. Conversely, CXCL‐1, CXCL‐2, and G‐CSF were shown to be expressed mainly by AM in the initial phase of infection (around 6 h post‐infection). Their results also show that the lack of CXCL5 expression leads to a temporary reduction in neutrophil infiltration in the lungs of infected mice. These observations are in agreement with our work since AIRmin mice were unable to express CXCL5 in response to pneumococcal infection and showed reduced neutrophil infiltration. Although the results of Yamamoto and collaborators did not associate the lack of CXCL5 expression with susceptibility to pneumococcal infection, the results observed in AIRmin mice indicate that the absence of this chemokine may have contributed, at least in part, to the susceptibility of these mice 34, 35.

MMPs participate in several aspects of inflammation, including extracellular matrix degradation, cell recruitment through the regulation of chemokines activities as well as regulation of cytokines activities. Increased susceptibility to pneumococcal infection was described for MMP2 and MMP9 double knockout mice. *Mmp2/9^−/−^* mice showed higher bacterial burdens when compared to WT mice and increased levels of IL‐17, IP‐10, and RANTES in the lungs 36, similar to the observed for AIRmin mice at 48 h post‐infection. When we analyzed the expression of these and other MMPs, we observed that the pneumococcal infection indeed induced the expression of MMP‐2, MMP‐3, MMP‐8 and pro‐MMP9 in the respiratory tract of AIRmax mice. Conversely, the levels of MMP‐2 and MMP‐3 remained unchanged in the airways of AIRmin mice whereas a delay in the secretion of MMP‐8 and pro‐MMP9 was observed. Results were much more complex when we measured enzymatic activity. Significant higher enzymatic activity upon the Abz‐AGLA‐EDDnp substrate was detected at 6 h but not at 12 h post‐challenge in BALF from AIRmax when compared to AIRmin mice. These results suggest that other MMPs may be present in the environment and may affect the overall response. Furthermore, although the substrate used satisfies the requirements of primary specificity, it may have different susceptibilities to hydrolysis by the MMPs of interest. Finally, although the results of in vivo inhibition of MMPs may be difficult to interpret, due to the difficulty to achieve effective inhibition at the site of infection, we have observed that treatment of AIRmax mice with MMP inhibitors, particularly SB‐3CT, altered the capacity of these animals to clear bacteria. Thus, our results support a role of MMPs in pneumococcal infection, firstly observed by the use of knockout mice. Different studies have already shown that inhibition or deficiency in MMP‐2, MMP‐3, MMP‐8, or MMP‐9, can lead to a reduction in neutrophil recruitment to inflammatory sites (revised by Ref. 37, 38). This is also in accordance to our observation that AIRmin mice have impaired expression of these MMPs and present reduced recruitment of neutrophils to the airways after pneumococcal infection.

Since very few AIRmax mice were not able to control infection at 48 h, they could not be included in our analysis of immune responses. However, it is important to mention that these animals showed high numbers of neutrophils and high levels of cytokines, chemokines, and MMPs at 48 h post‐infection indicating an exacerbated response at this time point. The profile was similar to the observed for AIRmin mice, but a massive inflammatory response was detected in these few mice at 48 h post‐infection (data not shown).

Apoptosis of AM has been shown to be an important mechanism for bacterial clearance. In addition, elimination of apoptotic AM through phagocytosis decreases inflammatory responses through the down‐regulation of TNF‐α, and the reduction in neutrophil recruitment, limiting tissue damage, and bacteremia 6, 7, 39. Although similar percentages of macrophages in apoptosis were observed at 12 and 48 h post‐infection for both AIRmin and AIRmax mice, higher percentages of macrophages in necrosis (late apoptosis) were observed in AIRmin mice at 48h post‐infection. Based on our results, two hypotheses could be proposed. AIRmin macrophages undergoing necrosis have limited bacterial killing capacity, resulting in the increase in bacterial burden in the lungs observed at 48 h. Alternatively, accumulation of necrotic macrophages, resulting in continuous inflammatory responses, as observed by the sustained levels of inflammatory mediators, could lead to tissue damage and bacteremia. Finally, AIRmin mice also showed higher percentages of neutrophils in both apoptosis and necrosis, when compared to AIRmax mice, at 48 h post‐infection. Neutrophils of *Mmp9* ^−/−^ mice were shown to have impaired bactericidal activity and viability, due to increased cell death 36. The delay in pro‐MMP‐9 secretion could also contribute to a similar effect in the response of AIRmin mice.

In an approach to evaluate the influence of host‐pathogen interactions on pneumococcal infection, Jonczyk and collaborators have followed the infection of BALB/c and CBA/c mice with ten pneumococcal strains. They observed that the pulmonary cytokine profile (and not single inflammatory mediators) at 6 and 24h post‐infection could discriminate between survivors and fatalities 40. In our approach, using outbred mouse stocks, only reduced secretion of CXCL5, a chemokine previously shown to be produced by EC during pneumococcal infection, could be related with the increased susceptibility of AIRmin mice to pneumococcal infection. In addition, impaired expression and activity of MMPs, known to regulate the activity of cytokines and chemokines and cellular influx, were observed in susceptible mice, adding another level of complexity in the inflammatory responses to pneumococcal infection.

## Conflict of Interest

The authors have no conflict of interest.

## Supporting information

Additional supporting information may be found in the online version of this article at the publisher's web‐site.

**Figure S1**. Pneumococcal nasal colonization of AIRmin and AIRmax mice. Animals were submitted to intranasal pneumococcal challenge with the serotype 6B 0603 strain.
**Figure S2**. Strategy for the analysis of cell populations in BALF. Animals were submitted to intranasal pneumococcal challenge with the serotype 3 ATCC6303 strain.
**Figure S3**. Cytokines and chemokines were induced in response to pneumococcal challenge in both AIRmin and AIRmax mice.
