# Supplementary material for: Impaired expression of CXCL5 and matrix metalloproteinases in the lungs of mice with high susceptibility to Streptococcus pneumoniae infection
Source: Immun Inflamm Dis. 2017 Nov 9;6(1):128–42. doi: 10.1002/iid3.205 (PMC5818448; doi:10.1002/iid3.205)
Supplement: Supplementary file 1 — Figure S1. Pneumococcal nasal colonization of AIRmin and AIRmax mice. Animals were submitted to intranasal pneumococcal challenge with the serotype 6B 0603 strain. Figure S2. Strategy for the analysis of cell populations in BALF. Animals were submitted to intranasal pneumococcal challenge with the serotype 3 ATCC6303 strain. Figure S3. Cytokines and chemokines were induced in response to pneumococcal challenge in both AIRmin and AIRmax mice. [file IID3-6-128-s001.docx]

**Pneumococcal nasal colonization challenge and recovery of bacteria from nasal washes.** Animals were anesthetized through the i.p. route with 20mg/Kg of xylazine and 25mg/Kg of ketamine and received 5x10^6^ CFU of the 0603 (serotype 6B) pneumococcal strain, in 10 µl of saline, inoculated in both nostrils with the help of a micropipette. Animals were euthanized 5 or 7 days after the inoculation with 60mg/Kg of xylazine and 300mg/Kg of ketamine for collection of Nasal washes as previously described [[1](#_ENREF_1)]. Serial dilutions of the samples were plated on blood agar containing 4μg/mL of gentamicin and CFU counting was performed after overnight incubation at 37 ºC. The minimal limit of detection was 40 CFU

**References**

**1. Hernani Mde L, Ferreira PC, Ferreira DM, Miyaji EN, Ho PL, et al. (2011) Nasal immunization of mice with Lactobacillus casei expressing the pneumococcal surface protein C primes the immune system and decreases pneumococcal nasopharyngeal colonization in mice. FEMS Immunol Med Microbiol 62: 263-272.**

**Figure S1.** Pneumococcal nasal colonization of AIRmin and AIRmax mice. Animals were submitted to intranasal pneumococcal challenge with the serotype 6B 0603 strain. Mice were euthanized 5 or 7 days after the challenge and bacteria were recovered from nasal washes. Circles represent each individual and lines represent the medians of the groups. Data were analyzed by Unpaired *T* test.


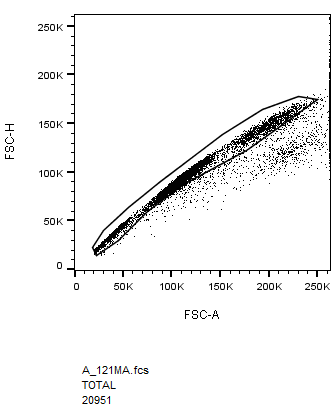

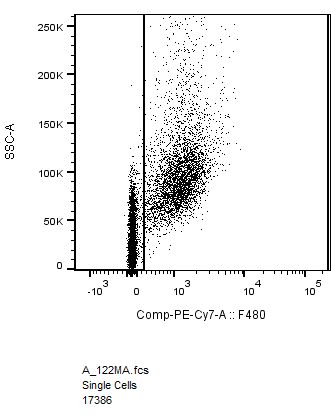

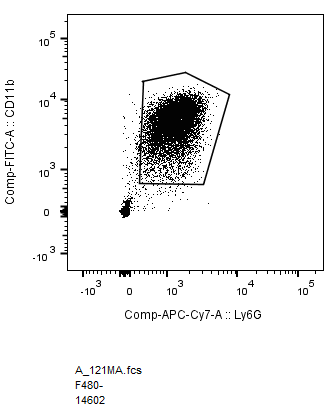

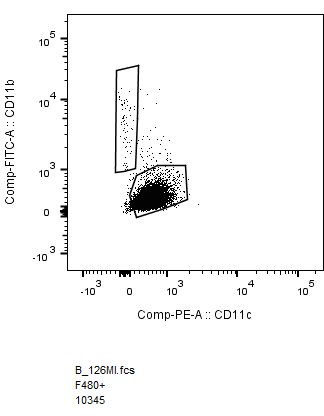

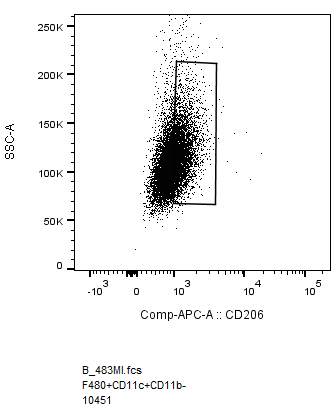


**FSC-A**

**FSC-H**

**F4/80**

**SSC-A**

**Ly6G**

**CD11b**

**CD11c**

**CD11b**

**CD206**

**SSC-A**

F4/80^-^

F4/80^+^

Neutrophils

(F4/80^-^ Ly6G^+^ CD11b^+^)

Interstitial Macrophages

(F4/80^+^ CD11b^+^ CD11c^-^)

Alveolar Macrophages

(F4/80^+^ CD11c^+^ CD11b^-^)

Alveolar Macrophages

(F4/80^+^ CD11c^+^ CD11b^-^ CD206^+^)

**Figure S2.** Strategy for the analysis of cell populations in BALF. Animals were submitted to intranasal pneumococcal challenge with the serotype 3 ATCC6303 strain. Mice were euthanized at different time points after the challenge and total cells from BALF were stained for the evaluation of neutrophils (F4/80^-^ CD11b^+^ Ly6G^+^), alveolar (F4/80^+^ CD11c^+^ CD11b^-^) or interstitial (F4/80^+^ CD11b^+^ CD11c^-^) macrophages. Expression of surface molecules in alveolar macrophages, such as the CD206 mannose receptor, was also analyzed. Positive subpopulations were gated based on the Fluorescence Minus One (FMO) strategy

**Figure S3.** Cytokines and chemokines were induced in response to pneumococcal challenge in both AIRmin and AIRmax mice. Animals (4 to 6 per group) were submitted to intranasal pneumococcal challenge with the serotype 3 ATCC6303 strain. Mice were euthanized at different time points after the challenge and BALF were collected for the analysis of cytokines and chemokines by Luminex. Results were expressed by means for each group with the standard deviations and are representative of two independent experiments. Data were analyzed by Two-way ANOVA with Tukey’s post-test, *P<0.05; **P<0.01; ***P<0.001.
